# Modelling environmental drivers of black band disease outbreaks in populations of foliose corals in the genus *Montipora*

**DOI:** 10.7717/peerj.3438

**Published:** 2017-06-12

**Authors:** Carla C.M. Chen, David G. Bourne, Christopher C. Drovandi, Kerrie Mengersen, Bette L. Willis, M. Julian Caley, Yui Sato

**Affiliations:** 1 Australian Institute of Marine Science, Townsville, QLD, Australia; 2 ARC Centre of Excellence for Mathematical & Statistical Frontiers, Queensland University of Technology, Brisbane, QLD, Australia; 3 College of Science and Engineering, James Cook University, Townsville, QLD, Australia; 4 School of Mathematical Sciences, Queensland University of Technology, Brisbane, QLD, Australia; 5 ARC Centre of Excellence for Coral Reef Studies, College of Science and Engineering, James Cook University, Townsville, QLD, Australia

**Keywords:** Black Band Disease, Environmental covariates, Coral Disease, Cyanobacterial patches, Transitional probability, Multi-state Markov model, Seasonal variation

## Abstract

Seawater temperature anomalies associated with warming climate have been linked to increases in coral disease outbreaks that have contributed to coral reef declines globally. However, little is known about how seasonal scale variations in environmental factors influence disease dynamics at the level of individual coral colonies. In this study, we applied a multi-state Markov model (MSM) to investigate the dynamics of black band disease (BBD) developing from apparently healthy corals and/or a precursor-stage, termed ‘cyanobacterial patches’ (CP), in relation to seasonal variation in light and seawater temperature at two reef sites around Pelorus Island in the central sector of the Great Barrier Reef. The model predicted that the proportion of colonies transitioning from BBD to Healthy states within three months was approximately 57%, but 5.6% of BBD cases resulted in whole colony mortality. According to our modelling, healthy coral colonies were more susceptible to BBD during summer months when light levels were at their maxima and seawater temperatures were either rising or at their maxima. In contrast, CP mostly occurred during spring, when both light and seawater temperatures were rising. This suggests that environmental drivers for healthy coral colonies transitioning into a CP state are different from those driving transitions into BBD. Our model predicts that (1) the transition from healthy to CP state is best explained by increasing light, (2) the transition between Healthy to BBD occurs more frequently from early to late summer, (3) 20% of CP infected corals developed BBD, although light and temperature appeared to have limited impact on this state transition, and (4) the number of transitions from Healthy to BBD differed significantly between the two study sites, potentially reflecting differences in localised wave action regimes.

## Introduction

Coral disease has contributed to localised declines in coral cover and changes in benthic communities (Weil, Smith & Gil-Agudelo, 2006; Harvell et al., 2007). For example, *Acropora palmata* and *A. cervicornis* populations declined in most Caribbean and Gulf of Mexico habitats in part due to diseases, while *Orbicella spp.* populations have suffered region wide declines due to yellow band disease (Gil-Agudelo et al., 2004; Bruckner & Bruckner, 2006a; Bruckner & Bruckner, 2006b). In the US Virgin Islands, coral disease following a mass beaching event in 2005 resulted in more than a 50% decline in coral cover, and in some areas of the wider Caribbean, repeated outbreaks of white band disease resulted in benthic communities shifting from coral to macroalgae dominated communities (Antonius, 1981; Harvell et al., 2007). The impacts of coral disease on reefs in other regions are not as extensively documented, although outbreaks have been observed across the Indo-Pacific (Raymundo et al., 2005; Weil et al., 2012) and in some areas of Great Barrier Reef (GBR) (Willis, Page & Dinsdale, 2004; Page & Willis, 2006; Sato, Bourne & Willis, 2009; Haapkylä et al., 2010).

Black band disease (BBD) presents as a virulent lesion that infects corals at reef locations worldwide, including the Caribbean, Red Sea and Indo-Pacific (reviewed in Sato et al. (2016)). On the GBR, BBD is also one of the most widespread coral diseases (Page & Willis, 2006). It appears as a darkly pigmented microbial mat occurring as a band at the interface between apparently healthy coral tissue and freshly exposed skeleton. The BBD microbial mat consists of a polymicrobial consortium, composed of a dominant cyanobacterium, sulfate-reducing and sulfide-oxidizing bacteria, and other heterotopic microorganisms, which migrates across colonies killing the underlying coral tissues (Richardson, 2004; Miller & Richardson, 2011; Sato et al., 2016). Linear progression rates of the band of up to 2 cm per day have been reported in the Caribbean (Kuta & Richardson, 1997), although typically it progresses more slowly (average: 0.3 cm/day; Sutherland, Porter & Torres, 2004). The prevalence of BBD on coral reefs is generally low, with only 1–10% of colonies typically infected at any one time (Green & Bruckner, 2000). Outbreaks can occur however, such as observed in the Florida Keys National Marine Sanctuary in 1992, where more than 50% of colonies within a population of *Orbicella annularis* (formerly known as *Montastraea annularis*; Budd et al. (2012)) were infected with the disease (Kuta & Richardson, 1996). At one study site on the GBR, BBD infections on approximately 10% of colonies in an assemblage resulted in an average loss of 40% of coral tissue surface area, with colonies having a history of BBD infection being particularly susceptible to re-infection (Sato, Bourne & Willis, 2009). Therefore, even though BBD is potentially part of the natural ecology of coral assemblages (Page & Willis, 2006), an outbreak of BBD is capable of reshaping a coral community (Bruckner & Bruckner, 1997).

Environmental conditions, particularly seawater temperature and light irradiance, combined with demographic factors, such as host diversity and density, have all been linked to the prevalence of a number of different coral diseases (Harvell et al., 2007; Harvell et al., 2009). For BBD specifically, changes in seawater temperature are thought to be a major environmental driver (Antonius, 1981; Edmunds, 1991; Kuta & Richardson, 2002; Rodriguez & Croquer, 2008; Sato, Bourne & Willis, 2009). High seawater temperatures can influence the dynamics of coral diseases through increased pathogen abundance and/or virulence, and/or increased host susceptibility as a result of reduced immune capacity (Burge et al., 2014). However, reports that BBD occurs mostly on corals in shallow habitats and is often absent from highly turbid waters suggest that spatial variation in the occurrence of this disease may be governed by the response of the microbial community associated with the lesion, particularly the dominant cyanobacterium, to different light intensities (Kuta & Richardson, 2002; Page & Willis, 2006; Cróquer & Weil, 2008).

During a two and a half year field monitoring study at a site in the central region of Australia’s GBR, cyanobacterium-dominated, green-brown lesions termed cyanobacterial patches (CP) were identified as an early stage in the development of BBD lesions (Sato, Willis & Bourne, 2010). The microbial community of CP was dominated by a cyanobacterium closely related to *Blennothrix* and *Trichodesmium spp.,* whereas the BBD microbial community was predominately composed of an *Oscillatoria sp*.-related cyanobacterium (Sato, Willis & Bourne, 2010), currently classified as *Roseofilum reptotaenium* (Casamatta et al., 2012; Buerger et al., 2016). In approximately 19% of colonies that presented with CP (*n* = 262 colonies), the lesion on these colonies developed into visually characteristic BBD, although this percentage is likely to be an underestimate because of difficulties accessing the sites during the monitoring period. Although the exact mechanism by which CP transitions to BBD is still unknown, a pathogenesis model proposed by Sato et al. (2016) suggests that light and temperature are key drivers of this transition. A physiological experiment using cyanobacterial cultures suggests that as light levels decrease from seasonal maxima and seawater temperatures approach seasonal maxima, conditions became favourable for the BBD-dominant cyanobacterium to outcompete the CP-associated cyanobacterium, facilitating transitions within the microbial community (Glas et al., 2010; Sato et al., 2016).

Statistical methods for studying disease transitions are well established for many host-pathogen interactions, and multi-state Markov models (MSMs) are particularly suitable for describing processes whereby an individual progresses through different states in a disease continuum and for exploring the roles of covariates in the process. For example, MSMs have been widely used in studies of human diseases, such as HIV/AIDS (Gentleman et al., 1994; Aalen et al., 1997; Mathieu et al., 2005), breast cancer (Meier-Hirmer & Schumacher, 2013) and dementia (Joly et al., 2002), however the use of such models to describe coral disease transitions has yet to be explored. Here, we apply an MSM to describe the development of BBD in 355 coral colonies monitored on the inshore central GBR to examine how changes in seasonal environmental conditions, in particular temperature and light, influence transitions between Healthy, CP and BBD states. Specifically, we (1) model the effects of seasonal changes in temperature and light on progression of BBD lesions, (2) test conclusions of the pathogenesis model proposed by Sato et al. (2016), and (3) provide a case study for applying such model-based approaches to understand drivers of coral disease outbreaks.

## Materials and Methods

### Data collection

The dynamics of the coral diseases CP and BBD were monitored in two *Montipora spp.*- dominated coral assemblages between September 2006 and January 2009, at sites in the central GBR located at North-East (18°32.5′S, 146°30.0′E) and South–East (18°33.6′S, 146°30.1′E) Pelorus Island (Map of the location is in Fig. S3), as detailed in Sato, Bourne & Willis (2009) and Sato, Willis & Bourne (2010). The average sea water temperature and photosynthetically active radiation recorded at the Pelorus Island site in the summer were 28.6°C and 497.0 µmol m^−2^s^−1^ and reduced to 22.6°C and 333.04 µmol m^−2^s^−1^in the winter.

Data from this intensive field monitoring program were used to develop modelling approaches for assessing drivers of disease transitions within coral populations. Both sites have limited exposure to terrestrial run-off but are exposed to strong wave energy year-round caused by south-easterly trade winds. The site at NE Pelorus is relatively more protected from waves than the SE Pelorus site. At each site, three replicate 10 m × 10 m permanent quadrants were haphazardly placed 5–10 m apart between 2.5 and 3.0 m depth. In this study, only coral colonies had signs of BBD during the study period were followed. A total of 355 coral colonies were individually tagged and photographed (239 colonies from SE Pelorus; 116 colonies from NE Pelorus), and the state of each coral colony was recorded in repeated surveys between September 2006 and January 2009 (see Sato, Bourne & Willis, 2009; Sato, Willis & Bourne, 2010 for full details). Due to logistical limitations in accessing study sites caused by poor weather conditions, surveys were done at irregular intervals (i.e., at one to three month intervals). The data was collected under the Great Barrier Marine Park Authority permits (No. G09/31013.1 and G09/30237.1.2).

### Environmental data

**Figure 1:**
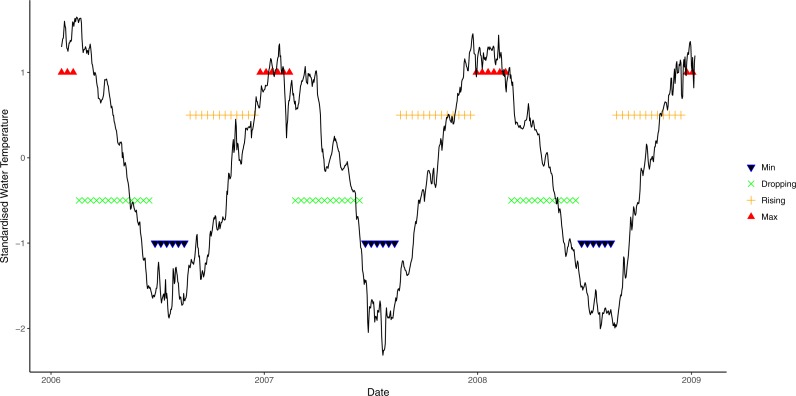
Seasonal variation in seawater temperature at 6 m from January 2006 to January 2009, showing four seasonal phases. Black line: daily mean temperature; blue lines: time period encompassing temperature minima; green lines: period when temperature is decreasing; orange lines: period when light is rising; and red lines: period when temperature at maxima.

Average daily seawater temperature and light irradiance levels were obtained from a weather station operated by the Australian Institute of Marine Science located at nearby Orpheus Island, approximately 8 km from the study sites. Seawater temperature was measured at 6 m depth and light at the surface as photosynthetically active radiation (PAR, µmol photons m^−2^s^−1^). As seawater temperature is a partial function of solar energy absorbed by the ocean, seasonal patterns of light and seawater temperature are highly correlated (Fig. S1). However, seasonal patterns in seawater temperature lag behind seasonal light patterns, thus light levels reach seasonal maxima/minima before seawater temperature. To incorporate the individual effects of both light and seawater temperature and account for the lag between the two variables, a new metric of environmental condition was developed by identifying four phases in annual light and seawater temperature cycles: rising (↑), maximum (M_ax_), declining (↓), and minimum (M_in_). To determine the seawater temperature phase at time *t*, a non-linear sinusoidal model was first fitted to each of the datasets. The water temperature phase at time *t* was then determined by the value of the slope of the non-linear function at point *t*, which is the first derivative of the function. Even though a slope of zero is the theoretical turning point of functions (i.e., slope = 0, either at the maximum or the minimum; slope > 0, rising phase; slope < 0, decreasing phase), a wider range of values was used here to reflect that water temperature often remains relatively steady for a period before declining or increasing. Exploratory analysis suggested that a threshold slope value of ≈0.7 best described the data. Therefore, when the slope was greater than 0.7, temperature was deemed to be rising, and decreasing when the slope was less than −0.7. Slopes between −0.7 and 0.7 were categorised as being either maxima or minima, depending on the observed value (Fig. 1).

**Figure 2:**
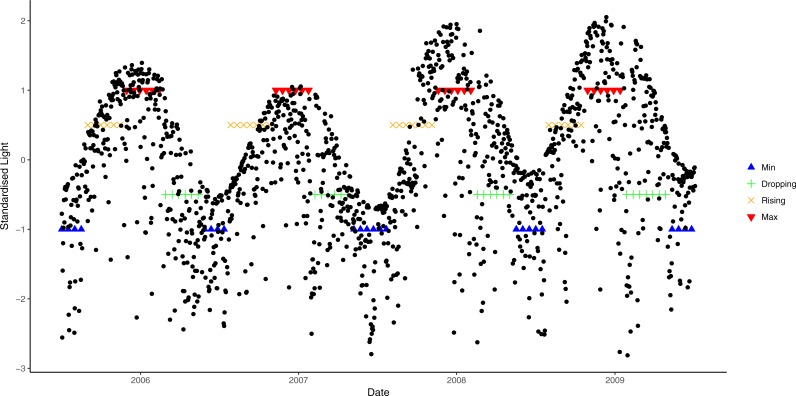
Seasonal variation in light from July 2006 to July 2009 and corresponding phases. Blue lines correspond to the light at trough, green lines are when light is at dropping phase, orange lines are when light is at rising phase and red lines are the light at crest.

A similar approach was used to derive light phases from daily average data. However, due to large annual variation in light cycles (Fig. 2), different cosine functions were fitted to each of the annual light cycles between July 2005 and July 2009. An annual light cycle was defined as 365 days starting from the lowest light period in July, and light data from July 2005 to July 2009 was used. Different threshold values were chosen for each annual cycle, based on the closest fit to natural patterns in an exploratory analysis (i.e., 0.9 for 2006, 0.8 for 2007 and 2008, and 0.7 for 2009; Fig. 2).

The converted categorical variables of light and seawater temperature were combined to form a single environmental metric using eight possible combinations (“M_ax_↑”: light at maxima and water temperature rising; “M_ax_M_ax_”: both light and water temperature at maxima; “↓M_ax_”: light dropping and water temperature at maxima; “↓↓”: both light and water temperature dropping; “M_in_↓”: light at minima and water temperature dropping; “M_in_M_in_”: both light and water temperature at minima; “↑M_in_”: light rising and water temperature at minima; and “↑↑”: both light and water temperature rising). However, due to the logistics of assessing study sites in poor weather conditions, only one observation for each of “↑M_in_”, “M_in_↓” and “↓M_ax_” phases was available. Therefore, samples from these phases were combined with the nearest class (by date), hence we used five possible phases of microclimatic conditions, “M_ax_M_ax_”, “M_ax_↑”, “↓↓”,“↑↑” and “M_in_M_in_”.

### Application of a multi-state model to explain CP-BBD disease transitions

A multi-state Markov model (MSM) was used to model transitions between disease states and refine environmental factors contributing to these transitions. This model is particularly useful when observations are made at irregular time intervals, the exact transition time is unknown, subjects are recruited progressively, and survival times are right censored (e.g., death of some subjects is not reached by the end of study). In a MSM, the probability of transition, i.e., moving from state *r* to state *s*, is governed by transitional intensity (*q*_*rs*_), which is the instantaneous risk of moving between two states (i.e., *r* to *s*), and the time interval between observations (*t*). When the effects of covariates are of interest, covariates are often regressed on the transitional intensity using the proportional hazard model, which assumes covariate effects are multiplicative, i.e., ${q}_{rs} \left( x \right) ={q}_{\mathit{rs}}^{(0)}\exp \left( {\beta }_{rs}x \right) $ where ${q}_{rs}^{(0)}$ is the baseline intensity and *β*_*rs*_ is the effect size of covariate *x*.

**Figure 3:**
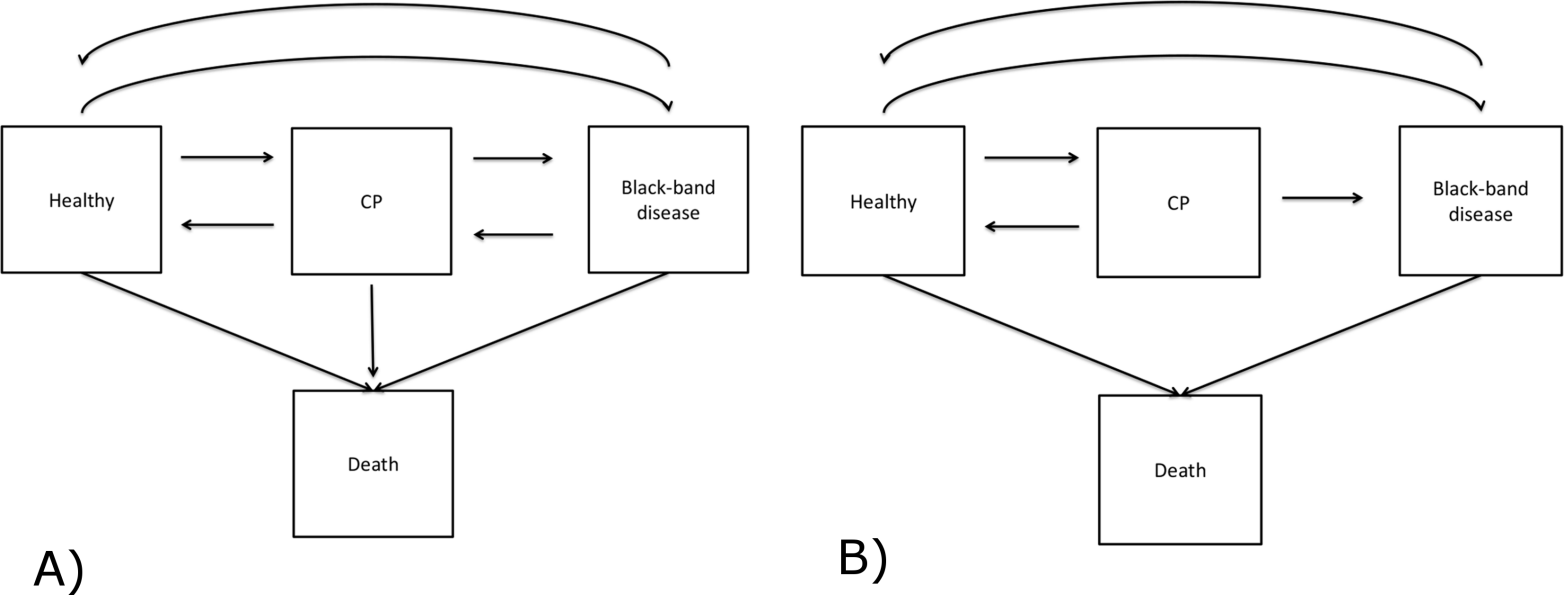
Modelling development of BBD lesions on the coral *Montipora* spp. Square boxes represent coral states and arrows denote the direction of disease development. Except for the death states, transitions between transient states are bi-directional. (A) is the initial disease model, and (B) is the final model implemented in the analysis.

We used a time-homogeneous Markov model to explain the development of BBD lesions. This model assumes the transition intensity is constant as a function of time, *t*, and independent of the history of the process, but only dependent on the state that the coral currently occupies. The time unit here is a month and a detailed description of the model is available in Supplemental Information 1. In our study, BBD disease development is specified to have four discrete states, including three transient states (Healthy, CP and BBD) and one absorbing state (Dead) (Fig. 3A). The healthy state was defined as a colony lacking any visual signs of CP or BBD lesions when examined. This state included colonies that showed no disease signs, although they may have had a lesion previously that has since disappeared. Death was defined here as mortality of an entire coral colony. To investigate the effects of light and water temperature conditions on the transition between healthy to diseased states, recovery, and between two diseased states, covariates were applied to transitions between H → CP, CP → H, CP → BBD, H → BBD and BBD → H. In addition to light and seawater temperature phase, study sites (NE and SE Pelorus) and disease density were also included as covariates. Disease density was defined as the number of infected coral colonies per 100 m^2^ quadrat at the time observed. Disease density was then categorized as: low (≤10 colonies), median (11–20 colonies) or high (≥21 colonies). These thresholds were selected based on the density of observed diseased colonies over the sampling period. The maximum disease density during the study period was 33 coral colonies per 100 m^2^, and there were three distinct clusters in the disease density (Fig. S2). The ranges of clusters were 0–10, 10–20, and 20 +.

The *msm* package (Jackson, 2007) in R was used for model fitting. Parameter estimation was done using the Broyden–Fletcher–Goldfarb–Shanno (BFGS) algorithm. Likelihood ratio tests were used to prevent overfitting, and the assumption of time–homogenous transition intensity was examined using the method suggested by Kalbfleisch & Lawless (1985), which involves fitting a time-dependent model, i.e ${q}_{sr} \left( t \right) ={q}_{sr}{\mathrm{e}}^{\mathrm{\lambda }t}$ and testing if *λ* = 0. All statistical analyses were carried out in R (R Core Team, 2016). The data and code used in this manuscript is available via https://github.com/cewels/Modelling-environmental-drivers-of-BBD-outbreak.

## Results

Disease states of 239 and 116 colonies of *Montipora sp*. from SE and NE Pelorus reefs, respectively, were repeatedly recorded between September 2006 and January 2009 (17 observations per colony at SE Pelorus; 13 observations per colony at NE Pelorus). The median duration between two observations was 1.67 months (range: 0.33–3.76 months). During the study period, the mean coral densities for SE and NE Pelorus were 227 and 289 colonies per 100 m^2^, and the mean BBD densities for both sites were 3.14 and 3.94 colonies per 100 m^2^, respectively. Although the size of each individual colony was not recorded, the surface area of most colonies was over 100 cm^2^ at both study sites. The majority of corals within each assemblage remained in the Healthy state between observations, and 214 transitions from Healthy to the CP state and 166 direct transitions from Healthy to the BBD state were also observed (Table 1). Eleven colonies that had no visible signs of disease died during the study and the cause of their mortality could not be assigned. For corals with CP, 160 transitioned back to the Healthy state, 87 remained in the CP state and 43 progressed to BBD by the next survey. On only two occasions did corals in the CP state die without a BBD lesion being observed (Table 1), hence transitions from CP to death were omitted from the subsequent MSM analyses (Table 2). For corals displaying visible signs of BBD, 150 returned to a Healthy state, 116 remained in the BBD state, and 11 colonies died. The transition from BBD to CP was observed 5 times; however, these represented new CP lesions elsewhere on the host coral after the original BBD lesions had disappeared, indicating that these BBD lesions did not transition back to the CP stage. Therefore, the transition from the BBD to CP state was also excluded from the MSM analysis. The final disease model is shown in Fig. 3B. The difference between the log likelihood of the time-dependent and time-independent models was small (−2 log-likelihood are 4354.405 and 4376.467). Therefore, the assumption of a time-independent MSM appears to be justified.

**Table 1:** Total number of state transitions occurring between 5,030 pairs of consecutive observations from September 2006 to January 2009. The number in the bracket is the proportion of the transitions in all observations.

		To			
		Healthy	CP	BBD	Dead
From	Healthy	4,065 (0.81)	214 (0.043)	166 (0.03)	11 (0.002)
	CP	160 (0.03)	87 (0.017)	43 (0.01)	2 (0.0004)
	BBD	150 (0.03)	5 (0.001)	116 (0.02)	11 (0.002)

**Table 2:** The effect of light-water temperature phase on the transition between two states. The light-water temperature is a categorical variable, thus the magnitude of phase effect is estimated using odds ratio. For example, the transition from healthy to CP was 2.85 higher during the ↑↑ phase comparing to ↓↓ phase. The two columns on the right are the estimated 95% confidence interval of the estimated odds ratio. M_in_M_in_ and M_ax_M_ax_ symbolize phases when both light and water temperature are at minima and maxima, respectivly; ↑↑ and ↓↓ represent when both light and water temperature are rising and dropping, respectively; and M_ax_↑ represents when the light is at maxima while seawater temperatures are rising.

Transition	Light-water temperature phase	Odds Ratio	Lower 95% CI	Upper 95% CI
(A) Healthy → CP	M_in_M_in_∕↓↓	2.04	0.95	4.36
	↑↑∕↓↓	2.85	1.35	6.02
	M_ax_↑∕↓↓	3.56	1.66	7.62
	M_ax_M_ax_∕↓↓	1.29	0.36	4.67
(B) Healthy → BBD	M_in_M_in_∕↑↑	0.07	0.001	3.28
	M_ax_↑/↑↑	5.21	2.71	10.01
	M_ax_M_ax_∕↑↑	3.50	1.46	8.39
	↓↓∕↑↑	1.54	0.58	4.06
(C) CP → Healthy	M_in_M_in_∕↑↑	1.37	0.68	2.80
	M_ax_↑∕↑↑	3.36	1.92	5.89
	M_ax_M_ax_∕↑↑	7.43	2.98	18.54
	↓↓∕↑↑	3.67	1.39	9.91
(D) BBD → Healthy	M_in_M_in_∕↑↑	1.54	0.77	3.08
	M_ax_↑∕↑↑	8.27	4.37	15.67
	M_ax_M_ax_∕↑↑	4.10	2.33	7.24
	↓↓∕↑↑	0.01	0.000	31.96

The final model fit was significantly better than the model without covariates (LR = 392.546, DF = 17, *p* < 0.001), but the likelihood ratio test demonstrated that not all covariates influenced the transitions between all states. Site and light-temperature phases were important for transitions from H → BBD, however only light-temperature phases were important for the transition intensities of H → CP, CP → H, and BBD → H. Disease density did not significantly affect the transition between H → CP or H → BBD.

Transitions between healthy and disease states (CP or BBD) were affected by the light-temperature phases. During the period when temperature was rising and light was either rising or at its annual maximum (i.e., ↑↑ or M_ax_↑), the transition intensity from H → CP was significantly higher than the period when both light and temperature were in decline (↓↓; Table 2A); transition intensities from H → CP were low and did not differ significantly among ↓↓, M_in_M_in_ and M_ax_M_ax_ phases. This suggests that healthy coral colonies were more likely to be affected by CP during the spring. In contrast, transitions from H → BBD occurred more frequently later in the summer season. During the M_ax_↑ and M_ax_M_ax_ phases, healthy coral colonies were 5.21 and 3.5 times, respectively, more likely to be affected by BBD than during the ↑↑ phase (Table 2B). However, there was little difference in the transition intensities between the M_in_M_in_, ↑↑ and ↓↓ phases.

The MSM results also showed strong spatial variation between the NE and SE Pelorus sites. The estimated instantaneous transitions from H → BBD (q_H→BBD_) at NE Pelorus were 2.41 times (95% CI [1.67–3.50]) higher than at SE Pelorus. This suggests that healthy corals at NE Pelorus more frequently contracted BBD than corals at the SE Pelorus site.

After accounting for the effects of covariates, the estimated baseline monthly transition intensity, ${q}_{\mathrm{H}\rightarrow \mathrm{cp}}^{(0)}$, from healthy into CP (H → CP) was slightly higher than transitioning from Healthy to BBD (${q}_{\mathrm{H}\rightarrow \mathrm{BBD}}^{(0)}$) (${q}_{\mathrm{H}\rightarrow \mathrm{cp}}^{ \left( 0 \right) }$, mean monthly transition intensity from H to CP was 0.05, 95% CI [0.040–0.065]; ${q}_{\mathrm{H}\rightarrow \mathrm{BBD}}^{ \left( 0 \right) }$, mean monthly transition intensity from H to BBD was 0.019, 95% CI [0.007–0.046], Table 3), however this difference was not significant. This suggested that without the influence of site and light-temperature phase, at any given time interval, the probability for H → CP is similar to H → BBD.

**Table 3:** Estimated baseline monthly transitional intensity, ${\mathbi{q}}_{rs}^{(0)}$ (instantaneous probability of transitioning from state *r* to *s* in a month) and 95% confidence interval between two included states. These are monthly transitional intensities without the effect of other covariates. For example, the monthly transition from CP to BBD was significantly lower than the transition from CP to Healthy, as the mean estimates were 0.19 (95% CI [0.132–0.274]) and 0.68 (95% CI [0.51–0.905]), respectively.

Transition	Mean estimates	Lower 95% CI	Upper 95% CI
Healthy → CP	0.051	0.04	0.065
Healthy → BBD	0.019	0.007	0.046
Healthy → Death	0.001	0.0003	0.003
CP → Healthy	0.680	0.51	0.905
CP → BBD	0.190	0.132	0.274
BBD → Healthy	0.301	0.086	1.04
BBD → Death	0.036	0.020	0.067

Even though the transition from CP to healthy predominately occurred during the M_ax_↑, M_ax_M_ax_ and ↓↓ phases (Table 2C), the effects of differing light-seawater temperature phases on this transition was less clear. This was because a higher number of HCP transitions occurred in the ↑↑ phase (period immediately before M_ax_↑ phase) and the estimated mean sojourn time for CP (i.e., the time remaining in the CP state) was 1.14 months (95% CI [0.91–1.43]). Therefore, it is unclear if the higher number of CP → H transitions was the result of light-temperature conditions or the development of a host immune response to the disease. Similarly, we were unable to identify the effect of light-seawater temperature phases on the transition from BBD → H, even though a high number of transitions from BBD → H were observed during the M_ax_↑ and M_ax_M_ax_ phases (Table 2D), as the estimated mean sojourn time for BBD was 2.67 months (95% CI [0.98–7.34]). Furthermore, we found no significant difference in the observed sojourn times of BBD among different light-seawater temperature phases (Likelihood ratio test of models with and without the inclusion of light-seawater temperature phases, test statistic = 2.17, *DF* = 4, *p* = 0.704), suggesting that reverting to the healthy state is likely due to the development of a host immune response to the disease.

After removing the covariate effect, coral colonies with CP were approximately four times more likely to revert to a healthy state than progress into the BBD state (monthly transition intensities for CP → H and CP → BBD were ${q}_{\mathrm{cp}\rightarrow \mathrm{H}}^{(0)}=0.68$ vs. ${q}_{\mathrm{cp}\rightarrow \mathrm{BBD}}^{(0)}0.19$, Table 3. The ratio of two intensities is approximately 4). Once a coral colony exhibited a BBD lesion, the estimated probability of recovery (BBD → H) in three months was approximately 53%. However, once a coral colony presented BBD, mortality was at least 30 times higher than a healthy colony (${q}_{\mathrm{BBD}\rightarrow D}^{(0)}=0.036$ v.s ${q}_{\mathrm{H}\rightarrow D}^{(0)}=0.001$; Table 3).

## Discussion

This study demonstrates the use of a multi-state analysis to understand the dynamics of a BBD disease within a *Montipora spp.* coral assemblage and elucidate how the covariate effects of light and temperature influence lesion state-transitions within individual colonies. Results highlight that the combined effect of seasonal variation in light and seawater temperature is an important driver for transitions of individual healthy *Montipora sp.* corals into either CP or BBD disease states. The transition into each of the two disease states occurred mostly from spring to summer, when light and seawater temperatures were rising or at their maxima (↑↑, M_ax_↑, M_ax_M_ax_). The transition between H → CP occurred slightly earlier in the spring/summer season than H → BBD, suggesting that CP may act as a precursor to BBD infections in some cases, although CP was more likely to heal (CP → H) than transition to BBD, as found in a field study (Sato, Willis & Bourne, 2010). Overall, healthy corals were more likely to develop CP lesions than BBD lesions, and the likelihood of CP developing was greater during spring when seawater temperatures and light were increasing or at their maxima (↑↑, M_ax_↑), compared to autumn months when temperature and light were declining (↓↓). The transition from healthy to CP subsided when light and temperature both reached maxima (i.e., M_ax_M_ax_), suggesting that rising seawater temperatures are favourable for the development of CP lesions but high temperatures above a certain threshold inhibited development of CP lesions. This interpretation is supported by laboratory-based studies, which found that high temperatures at summer maxima negatively affected growth of the dominant cyanobacterium within CP lesions (Glas et al., 2010). Thus, lower growth rates of the dominant cyanobacterium within CP lesions likely explains the lower probability of CP-development when both light and temperature were at maxima. In contrast, evidence that growth of CP-derived cyanobacteria in cultures was positively correlated with light intensity (Glas et al., 2010) explains why the highest intensity of H → CP transitions occurred when light was at its maximum (M_ax_↑).

The transition intensity between healthy and BBD states peaked when light was at its maximum and water temperature was rising or at its maximum (M_ax_↑, M_ax_M_ax_). The 3–5 times greater probability of developing BBD during the M_ax_↑ andM_ax_M_ax_ phases than when both light and temperature were rising (↑↑) suggests that certain light and potentially temperature thresholds need to be reached before corals are susceptible to BBD. Previous field studies have showed that BBD abundance is positively correlated with temperature and light intensity (Antonius, 1981; Edmunds, 1991; Kuta & Richardson, 2002; Page & Willis, 2006; Voss & Richardson, 2006; Weil & Cróquer, 2008; Sato, Bourne & Willis, 2009; Zvuloni et al., 2009; Muller & Van Woesik, 2011). Culture-based studies of the locally dominant cyanobacterium in BBD lesions show that its growth is enhanced at seasonal temperature maxima, while light has little impact on its growth (Glas et al., 2010), corroborating our field-based results. Aquarium-based experimental studies have also shown that both high light and temperature can cause stress in coral hosts and are linked to an increase in BBD virulence (Boyett, Bourne & Willis, 2007; Sato, Bourne & Willis, 2011). Furthermore, a recent metagenomic and metatranscriptomic-based study on the *in-situ* development of BBD derived from CP showed that increased cyanobacterial photosynthesis, which introduces fixed carbohydrates into the microbial community, is a key to the development of BBD pathogenesis (Sato et al., 2017). However, our modelling approach did not detect a role for combined light and temperature variation to drive the CP to BBD transition. However, this result is likely due to the small number of CP-infected colonies developing into BBD (43 cases). Hence more observations are required to help elucidate the impact of light and temperature on the transition between the two disease states.

In addition to seasonal variation, our results also suggest strong spatial variation in the likelihood that colonies of *Montipora* transition from healthy to BBD states. Significantly more transitions from healthy to BBD were recorded at the NE than the SE Pelorus site. Considering that the distance between these two sites is less than 5 km, the difference in BBD susceptibility is likely to reflect localised environmental conditions, particularly differences in local wave action. Reefs at SE Pelorus are typically exposed to high wave action, whereas the NE Pelorus site is comparatively protected by a local headland. Constant surface disturbances and turbidity caused by wave surge would reduce light intensity reaching the reef substratum, thus light levels may regularly be lower at SE than at NE Pelorus, accounting for differences in disease dynamics between the two sites.

The low explanatory power of BBD-infected coral density on the probability of BBD development suggests that environmental factors are more important drivers of disease occurrence than the density of potential pathogen sources. Evidence of spatial clumping of BBD-infected corals in past monitoring studies led to the proposal that BBD spreads from infected corals to new corals in a density-dependant manner (Bruckner, Bruckner & Williams, 1997; Page & Willis, 2006; Voss & Richardson, 2006). In contrast, Edmunds (1991) reported that distributions of BBD-infected corals were not clumped nor dependant on host-coral density, suggesting that BBD is not highly contagious. The present study supports this latter hypothesis and suggests that the clumped distribution of BBD may result from patchy distributions of other local environmental conditions within reefs, such as bottom topology, light availability, and/or sedimentation rates.

MSM has commonly been used in medical research to understand the development of human diseases. Our application of this approach to the dynamics of the virulent coral disease BBD, specifically the effect of environmental covariates on the probability of transitioning between healthy and disease states in *Montipora spp.,* provides empirical support for the light-seawater temperature hypothesis established in Sato et al. (2016). Although this study would have benefited from a longer time series of observations made at shorter time-intervals, as well as more comprehensive and localised measurements of environmental covariates at each study site, it does provide a model-based framework for identifying the drivers of disease transitions at fine spatial and temporal resolution. As the frequency of disease outbreaks is predicted to increase with global changes in climate (Maynard et al., 2015), identifying the drivers of finer spatial and temporal heterogeneity of disease outbreaks and spread is becoming important, particularly for understanding the resilience of corals to climate change. Our findings provide novel insights into disease dynamics at the scale of individual coral colonies and identify environmental drivers leading to development of CP and BBD lesions on corals.

##  Supplemental Information

10.7717/peerj.3438/supp-1 Supplemental Information 1 Supplementary Method

10.7717/peerj.3438/supp-2 Supplemental Information 2 Supplementary Material

## Additional Information and Declarations

### Field Study Permissions

The following information was supplied relating to field study approvals (i.e., approving body and any reference numbers): Field experiments were approved by the great barrier Reef Marine Park Authority permit number G09/31013.1 and G09/30237.1.2.

### Data Availability

The following information was supplied regarding data availability: The data and code used in this study and the description of variables are now available via Github: https://github.com/cewels/Modelling-environmental-drivers-of-BBD-outbreak.
