# Supplementary material for: Modelling environmental drivers of black band disease outbreaks in populations of foliose corals in the genus Montipora
Source: PeerJ. 2017 Jun 12;5:e3438. doi: 10.7717/peerj.3438 (PMC5470580; doi:10.7717/peerj.3438)
Supplement: Supplemental Information 1 [file peerj-05-3438-s001.docx]

**Supplementary Method**

**Further details on the Multistate Markov model**

The Multistate Markov model assumes a finite number of states,$\mathcal{S=}\left\{ 1, \ldots, R \right\}$. The transition between the states is governed by a continuous time stochastic process $x\left( t \right)$ which takes values in $\mathcal{S}$. $H_{t}^{-}$ denotes the history of the stochastic process up to time $t$ and the probability of transitioning from state *r* to state *s* is;

$\text{p}_{rs}\left( t_{1}, t_{2} \right)\text{=}\text{p}\left( \mathcal{x}\left( t_{2} \right)=s | \mathcal{x}\left( t_{1} \right)\text{=}r\text{,} H_{t}^{-} \right)$.

The transition probability, $\text{p}_{rs}\left( t_{1}, t_{2} \right)$, is governed by the *transition intensity* $q_{rs}$, which is the instantaneous hazard rate of moving from state *r* to state *s* and is determined using the equation;

$$q_{\text{rs}}(\delta t, H_{t}^{-})=\lim_{\delta t\to0}\frac{p(\mathcal{x(}t_{1}+\delta t)=\text{s}\mathcal{|x(}t_{1})=r, H_{t}^{-})}{\delta t}$$

The effect of covariates are then included in the model through the intensity matrix;

$$q_{rs}(\delta t, H_{t}^{-})=q_{rs}^{0}\left( t, H_{t}^{-} \right)exp(\boldsymbol{\beta}_{rs}^{T}\boldsymbol{z})$$

where $q_{rs}^{0}\left( t, H_{t}^{-} \right)$ is the baseline intensity at time *t*, **z** is the covariate vector and $\boldsymbol{\beta}_{rs}$ is the vector of the effect of covariates for transition from state *r* to *s*.

Instead of using the entire transition history up to time *t*, the simplest approach is to assume the stochastic process, $x(.)$, has the *Markov* property, i.e. the future state is conditionally independent of the past history, given the current state. Therefore,

$$q_{rs}(\delta t)=\lim_{\delta t\to0}\frac{p(\mathcal{x(}t_{1}+\delta t)=s\mathcal{|x(}t_{1})=r)}{\delta t}$$

and the effect of the covariate is accommodated by

$q_{rs}(\delta t)=q_{rs}^{0}\left( t \right)exp(\boldsymbol{\beta}_{rs}^{T}\boldsymbol{z})$.

The other assumption of the stochastic process is that the transition intensity is homogenous. The transition probability, $\text{p}_{rs}\left( t_{1}, t_{2} \right),$ depends only on the interval of $t_{2}$ and $t_{1}$, thus

$$p_{rs}\left( t \right)=\exp\left( tq_{rs} \right)$$

where $t=t_{2}-t_{1}$.

*Sojourn time* describes the time occupying a single state *r,* which often is an interest in survival analysis studies. In the time-homogenous Markov model, the sojourn time is assumed to have an exponential distribution with mean $1/q_{rr}$.

Different assumptions can be made about the dependency between transition rate and time.


Meira-Machado et al.


(2008) has a detailed description of different assumptions and associated models.

**Comparing groups and transitions**

In survival analysis, the transition intensities are often referred to as *hazards*. The ratio of two hazards (i.e. hazard ratio) is commonly used for comparison of two groups in a covariate, for example treatment vs control


(Campbell et al. 2007). Given that the difficulty in identifying a control condition in a given light-temperature phase and that light-temperature phase impacts transitions differently, we could not identify any universal control for all transitions. Therefore, for each transition, we selected the phase with the least effect size as the control.

In this study, we also used the ratio of baseline transition intensities to compare the likelihood to transitioning into different states at a given state.

**Reference**

Campbell, M.J., Walters, S.J. & Machin, D., 2007. *Medical statistics*, John Wiley & Sons Ltd, West Sussex England.

Meira-Machado, L.F. et al., 2008. Multi-state models for the analysis of time-to-event data. *Statistical methods in medical research*. [18(2): 195–222.](https://www.ncbi.nlm.nih.gov/entrez/eutils/elink.fcgi?dbfrom=pubmed&retmode=ref&cmd=prlinks&id=18562394)
