# Supplementary material for: Modelling environmental drivers of black band disease outbreaks in populations of foliose corals in the genus Montipora
Source: PeerJ. 2017 Jun 12;5:e3438. doi: 10.7717/peerj.3438 (PMC5470580; doi:10.7717/peerj.3438)
Supplement: Supplemental Information 2 [file peerj-05-3438-s002.docx]

**Supplementary Figures**

1. **Correlation and time lag of water temperature at 6m and light irradiance**

Figure below shows the correlation between water temperature at 6m and light irradiance at the surface from January 2003 to June 2010 illustrating the clear time lag between the two factors as temperature consistently lags.

Figure 1 Light and sea water temperature from Jan 2003 to June 2010.

1. **Kernel Density of disease prevalence**

Figure 2 Three distinct clusters were observed in the density of disease prevalence. The ranges of each cluster were 0-10, 10-20 and 20+ colonies.

1. **Map of study site**

Figure 3 The map showing the location of study site. Pelorus Island is located in the central GBR with wave predominantly from the South-East.
